# Choroidal neovascularization associated with butterfly-shaped pattern dystrophy - a case report

**DOI:** 10.22336/rjo.2023.32

**Published:** 2023

**Authors:** Marta Świerczyńska, Marta Danikiewicz-Zagała, Lech Sedlak, Marta Nowak-Wąs, Dorota Wyględowska-Promieńska

**Affiliations:** * Department of Ophthalmology, Faculty of Medical Sciences in Katowice, Medical University of Silesia, Katowice, Poland; ** Department of Ophthalmology, Kornel Gibiński University Clinical Center, Medical University of Silesia, Katowice, Poland; *** Department of Ophthalmology, Euromedic Hospital, Katowice, Poland; **** Department of Histology and Cell Pathology, Faculty of Medical Sciences in Zabrze, Medical University of Silesia, Katowice, Poland

**Keywords:** butterfly-shaped pattern dystrophy, choroidal neovascularization, CNV, anti-VEGF treatment, ranibizumab

## Abstract

The pattern dystrophies (PDs) are a group of primarily autosomal dominant inherited macular diseases that cause the deposition of lipofuscin in retinal pigment epithelium (RPE) and may lead to significant vision loss in later life. Patients can develop choroidal neovascularization (CNV) and/ or geographic atrophy (GA) and for this reason they are often misdiagnosed as age-related macular degeneration (AMD). We presented a case of a 66-year-old patient complaining of vision loss in the right eye (RE) for 8 months. At the initial examination, his best corrected visual acuity (BCVA) was 0.6 in the RE. Optical coherence tomography angiography (OCTA), fundus autofluorescence (FAF) and fundus fluorescein angiography (FFA) allowed to diagnose butterfly-shaped PD in both eyes with choroidal neovascularization (CNV) in the RE. The patient was treated with three intravitreal anti-vascular epithelial growth factor (anti-VEGF, ranibizumab) injections during six weeks intervals, which improved and stabilized the BCVA of the RE to 0.7 during the over two-year observation period. Our report contributes to the still limited data regarding CNV associated with butterfly-shaped PDs and the results of treatment with ranibizumab.

**Abbreviations: **AMD = age-related macular degeneration, anti-VEGF = anti-vascular epithelial growth factor, AOFVD = adult-onset foveomacular vitelliform dystrophy, BCVA = best corrected visual acuity, CNV = choroidal neovascularization, FAF = fundus autofluorescence, FFA = fundus fluorescein angiography, GA = geographic atrophy, LE = left eye, MIDD = maternally inherited diabetes and deafness, OCT = optical coherence tomography, OCTA = optical coherence tomography angiography, OU = oculus uterque, both eyes, PD = pattern dystrophy, PDSFF = pattern dystrophy simulating fundus flavimaculatus, PDT = photodynamic therapy, PRPH2 = peripherine-2, RE = right eye, RPE = retinal pigment epithelium, VA = visual acuity

## Introduction

The pattern dystrophies (PDs) are a heterogenous group of primarily autosomal dominantly inherited macular diseases characterized by an abnormal accumulation of lipofuscin material at the level of the retinal pigment epithelium (RPE) caused by mutations in the peripherin 2 gene on chromosome 6p21.1 [**1**]. Moreover, PDs have been described in association with myotonic dystrophy, Kjellin syndrome, pseudoxanthoma elasticum, maternally inherited diabetes and deafness (MIDD) [**2**-**5**].

PDs are divided into 5 types based on the pattern of pigment distribution: 1) adult-onset foveomacular vitelliform dystrophy (AOFVD); 2) butterfly-shaped pattern dystrophy; 3) pattern dystrophy simulating fundus flavimaculatus (PDSFF); 4) reticular dystrophy of the RPE; 5) fundus pulverulentus [**6**]. PDs usually show bilateral and symmetrical involvement. However, the pattern distribution may be unilateral; each eye of the patient can have a distinct pattern, at different stages and the same mutation may correspond to various inter- and intrafamilial phenotypes [**7**]. 

The onset of symptoms usually occurs in the fourth or fifth decade and involve moderate vision loss and metamorphopsia. Some patients may remain asymptomatic throughout life, but PDs may lead to severe visual impairment due to atrophy of the RPE-photoreceptor complex, development of choroidal neovascularization (CNV) or macular holes as well [**1**]. 

This report describes the patient with CNV associated with butterfly-shaped PD. The use of intravitreal ranibizumab injections led to a remarkable improvement in VA, significant reduction of retinal thickness and a normalization of the macular appearance on optical coherence tomography (OCT) over a 26-month follow-up period.

## Case presentation

A 66-year-old Caucasian man presented to our clinic complaining of decreased visual acuity (VA) and metamorphopsias in his right eye (RE) for eight months. He was treated for glaucoma with latanoprost once a day in both eyes (OU) and had a mild stick injury around his left eye (LE) ten years before. His medical history included diabetes mellitus and hyperlipidemia. The family history was insignificant.

**Fig. 1:**
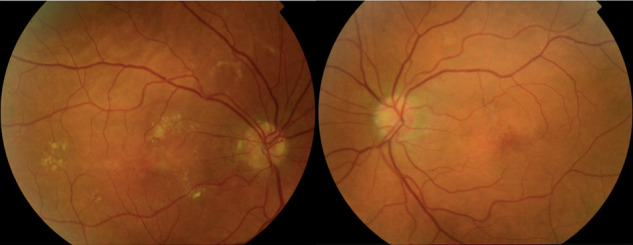
Color photographs of the right fundus: **A.** showed optic nerve head drusen, butterfly-like yellowish flecks, hard exudates, and angiopathy; **B.** optic nerve with slightly blurred margins and pigmentary changes in the macula were observed in the left eye (LE)

At the initial examination, his best-corrected visual acuity (BCVA) was 0.6 in the RE and 1.0 in the left eye (LE). The intraocular pressure (IOP) was 14 mmHg in the RE and 15 mmHg in the LE. The anterior segment examination revealed early signs of cataract OU and a scar with ingrown peripheral blood vessels in the left cornea. Fundus examination under mydriasis showed optic nerve head drusen, butterfly-like yellowish flecks, hard exudates and angiopathy in the RE, whereas in the LE, optic nerve with slightly blurred margins and pigmentary changes in the macula were noted (**Fig. 1 A, B**).

**Fig. 2:**
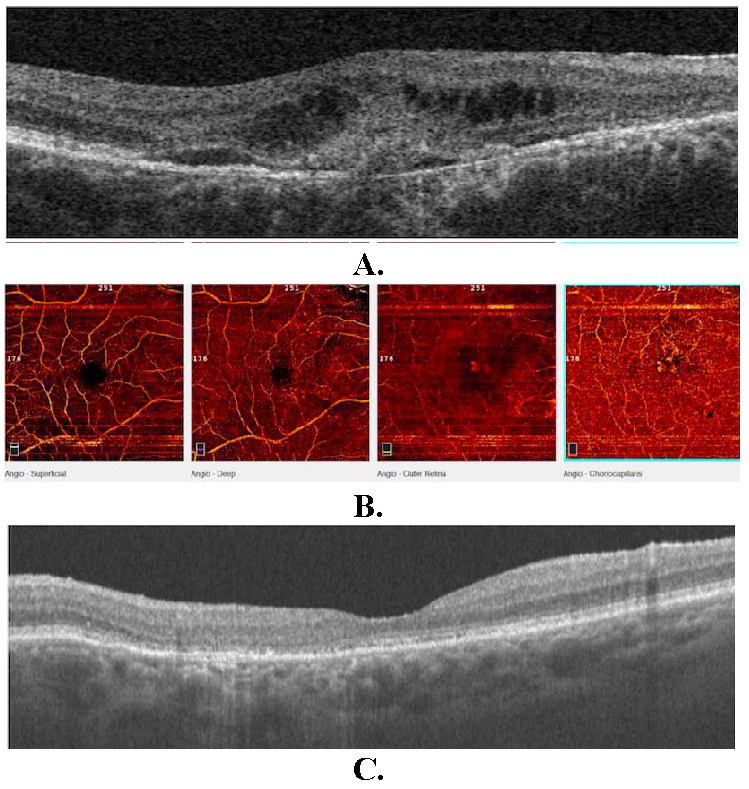
Optical coherence tomography (OCT) of the right eye (RE) at the initial examination **A.** revealed interruption of retinal pigment epithelium (RPE), subfoveal hyperreflective material with surrounding subretinal fluid, intraretinal cysts and increased retinal thickness. OCT angiography (OCTA) of the right eye (RE) **B.** allowed to identify choroidal neovascularization (CNV) in the outer retina and choriocapillaris layers of the macula. After 3 ranibizumab injections, OCT of the RE **C.** showed resolution of subretinal fluid and choroidal neovascularization (CNV), retinal thickness decreased as well

**Fig. 3:**
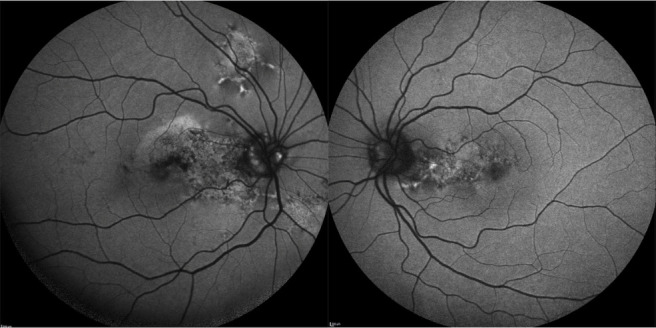
FAF demonstrated hyperautofluorescent flecks with adjacent zones of hypo-autofluorescence in the macula and papillomacular bundle area in both eyes. The lesions are also located above and below the optic disc, and the optic nerve head drusen are visible in the right eye (RE)

**Fig. 4:**
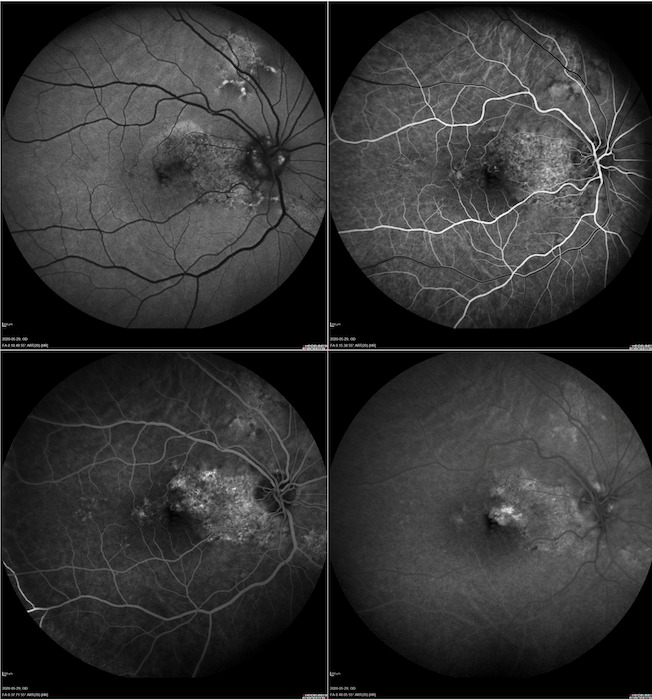
Fundus fluorescein angiography (FFA) of the right eye (RE) revealed small areas of hyperfluorescence staining corresponding to the flecks around the macula during early phases with normal choroidal flush - an area of increasing hyperautofluorescence in late phases suggestive of choroidal neovascularization (CNV) leakage

Optical coherence tomography (OCT) of the RE showed interruption of RPE, subfoveal hyperreflective material with surrounding subretinal fluid, intraretinal cysts, and increased retinal thickness. OCT of the LE revealed minor distortions in the RPE layer (**Fig. 2 A**). OCT angiography (OCTA) allowed to identify choroidal neovascularization (CNV) in the outer retina and choriocapillaris layers of the macula in the RE (**Fig. 2 B**). Fundus autofluorescence (FAF) demonstrated optic nerve head drusen and hyperautofluorescent flecks with adjacent zones of hypo-autofluorescence in the macula and papillomacular bundle area in both eyes, as well as above and below the optic disc in the RE allowing to confirm butterfly-shaped PD (**Fig. 3**). Fundus fluorescein angiography (FFA) revealed small areas of hyperfluorescence staining corresponding to flecks around the macula during early phases with normal choroidal flush; an area of increasing hyperautofluorescence in late phases suggestive of CNV leakage in the RE (**Fig. 4**). 

The patient was treated with intravitreal anti-vascular endothelial growth factor (anti-VEGF) injections - ranibizumab (Lucentis, Novartis, Switzerland) at six weeks intervals. After three injections, no evidence of subretinal or intraretinal fluid was observed in the OCT scans, and retinal thickness at the macula decreased (**Fig. 2 C**). At the end of the treatment course, BCVA improved to 0.7 in the RE and remained stable in subsequent follow up examinations over the next 26 months.

## Discussion

PDs are a group of congenital disorders caused by the mutation of the peripherin 2. Both autosomal dominant and recessive transmission have been observed and cases of incomplete penetrance or variable expressivity have been reported as well [**8**]. Peripherine-2 (PRPH2) is a transmembrane glycoprotein crucial for the formation, stabilization and renewal of the photoreceptor outer segment discs by acting as an adhesive element [**9**]. The main feature in PDs is accumulation of extracellular material beneath the sensory retina at the fovea due to malfunction of photoreceptor outer segment metabolism [**7**]. Although various gene mutations have been shown in PDs, the diagnosis can be made mainly with the aid of clinical examination and conventional imaging methods [**10**]. 

The clinical features that distinguish PDs from AMD include a relative earlier age of symptoms onset, the fundoscopic absence of the typical drusen and the presence of yellow-gray pigment in various patterns in the macula that show distinct changes on FAF imaging. However, later stages of PDs may resemble AMD and pigment deposits can resemble drusen. What is more, patients can develop CNV and/ or geographic atrophy (GA) and for this reason these cases are often misdiagnosed as AMD [**11**]. Central serous chorioretinopathy, Stargardt disease and Best disease should be included in the differential diagnosis [**12**].

The evolution of PD has been considered erroneously benign for a long time, probably due to frequent mistakes with more frequent and known macular diseases, above all AMD. It is postulated that 42% of patients with PDs will develop severe visual damage due to the atrophic (26%) or neovascular (18%) complications [**13**]. 

The precise mechanism of CNV development in PDs has not been established. Initially, it was hypothesized that the metabolic byproducts may accumulate after the RPE digests the mutant PRPH2 proteins, which may disrupt the extracellular matrix that helps maintain the barrier against CNV [**14**]. However, Casilo et al. [**15**] revealed that the PD subgroup showed thicker choroidal thickness both centrally and underneath the neovascular lesion in contradistinction to the AMD subgroup. Presumably, the thickening of the outer retinal vessels in the Haller layer could cause secondary damage to the overlying choriocapillaris network and induce ischemic phenomena responsible for neovascularization. Interestingly, one case of spontaneous regression of CNV in PD was observed [**16**].

Possible treatment options for CNV associated with PD include laser photocoagulation, photodynamic therapy (PDT) and anti-VEGF therapy. Previously described unexpected enlargement of the scar after laser photocoagulation of an extrafoveal CNV secondary to PD suggests that the PRE may be irreversibly damaged by the thermal insult [**17**]. What is more, PDT has been used to treat PD-related subfoveal CNV. This approach provided temporary advantageous effect on VA, which was followed by a progressive deterioration in the long-term. Probably, the effect of laser photocoagulation or PDT on primarily impaired RPE cells may lead to greater damage with consequent apoptosis [**18**].

On the contrary, it is documented in literature that due to non-AMD conditions, CNV respond better to anti-VEGF treatment and sometimes even one intravitreal anti-VEGF injection was satisfactory to control the CNV and stabilize VA [**19**,**20**]. Parodi et al. [**21**] reported a series of 12 patients with PD and subfoveal CNV, who were treated with course of intravitreal bevacizumab and documented a good anatomical and functional outcome. Nangia et al. [**22**] reported a case of CNV secondary to PD simulating fundus flavimaculatus, whereas Gallego-Pianzo et al. [**23**] published a series of six patients diagnosed with CNV associated with AOFVD. Successful visual effects after intravitreal ranibizumab treatment were described in both studies. Empeslidis et al. [**24**] documented the satisfactory results of the use of intravitreal ranibizumab in the treatment of a patient with CNV due to butterfly-shaped PD of the macula.

However, in the pilot study performed by Casillo et al. [**15**], a comparison between the long-term response to intravitreal ranibizumab among patients with PD-associated CNV and CNV in AMD, showed that eyes with PD had a poorer long-term response and greater tendency to atrophic evolution of the disease. Therefore, it is suggested that CNV associated with PD should not be “overtreated” to avoid further atrophic effects. One of the possible reasons for overtreatment lies in the hypothesis that part of the subretinal fluid could be due to RPE pump failure instead of CNV activity.

## Conclusions

Our report contributes to the still limited data regarding the results of anti-VEGF treatment use in CNV associated with butterfly-shaped PD. We achieved improvement and stabilization of anatomical and functional status after three ranibizumab intravitreal injections. It may support the fact that anti-VEGF treatment offers significant benefits in patients with PD-associated CNV. Nevertheless, the further observation of long-term effects and larger study group are required to further improve the understanding of butterfly-shaped PDs and their treatment.

**Conflict of Interest**

The authors declare that there are no conflicts of interests.

**Informed Consent and Human and Animal Rights statements**

The authors certify that they have obtained all appropriate patient consent forms. The patient has given his consent for his de-identified images and other clinical information to be used for teaching and research purposes. The patient understands that his name and initials will not be published and due efforts will be made to conceal the identity, but anonymity cannot be guaranteed. 

**Authorization for the use of human subjects**

Ethical approval: The research related to human use complies with all the relevant national regulations, institutional policies, is in accordance with the tenets of the Helsinki Declaration and has been approved by the institutional review board of the Ophthalmology Department, Kornel Gibiński University Clinical Center, Medical University of Silesia, Katowice, Poland.

**Acknowledgements**

None. 

**Sources of Funding**

None. 

**Disclosures**

None.

## References

[1] Zhang K, Garibaldi DC, Li Y, Green WR, Zack DJ (2002). Butterfly-Shaped Pattern Dystrophy: A Genetic, Clinical, and Histopathological Report. Arch Ophthalmol.

[2] Agarwal A, Patel P, Adkins T, Gass JD (2005). Spectrum of pattern dystrophy in pseudoxanthoma elasticum. Arch Ophthalmol.

[3] Massin P, Virally-Monod M, Vialettes B, Paques M, Gin H, Porokhov B (1999). Prevalence of macular pattern dystrophy in maternally inherited diabetes and deafness. GEDIAM Group. Ophthalmology.

[4] Kimizuka Y, Kiyosawa M, Tamai M, Takase S (1993). Retinal changes in myotonic dystrophy. Clinical and follow-up evaluation. Retina.

[5] Farmer SG, Longstreth WT Jr, Kalina RE, Todorov AB (1985). Fleck retina in Kjellin’s syndrome. Am J Ophthalmol.

[6] Gass JD (1997). Stereoscopic Atlas of Macular Diseases: Diagnosis and Treatment.

[7] Hannan SR, de Salvo G, Stinghe A, Shawkat F, Lotery AJ (2013). Common spectral domain OCT and electrophysiological findings in different pattern dystrophies. Br J Ophthalmol.

[8] Boon CJ, van Schooneveld MJ, den Hollander AI, van Lith-Verhoeven JJ, Zonneveld-Vrieling MN, Theelen T (2007). Mutations in the peripherin/RDS gene are an important cause of multifocal pattern dystrophy simulating STGD1/fundus flavimaculatus. Br J Ophthalmol.

[9] Farjo R, Naash MI (2006). The role of Rds in outer segment morphogenesis and human retinal disease. Ophthalmic Genet.

[10] Crane ER, Bass SJ (2019). Case Series: Multimodal Imaging Reveals the Spectrum of Pattern Dystrophies of the Retinal Pigment Epithelium. Optom Vis Sci.

[11] Saksens NT, Fleckenstein M, Schmitz-Valckenberg S, Holz FG, den Hollander AI, Keunen JE (2014). Macular dystrophies mimicking age-related macular degeneration. Prog Retin Eye Res.

[12] Ozkaya A, Garip R, Nur Tarakcioglu H, Alkin Z, Taskapili M (2018). Clinical and imaging findings of pattern dystrophy subtypes; Diagnostic errors and unnecessary treatment in clinical practice. J Fr Ophtalmol.

[13] Francis PJ, Schultz DW, Gregory AM, Schain MB, Barra R, Majewski J (2005). Genetic and phenotypic heterogeneity in pattern dystrophy. Br J Ophthalmol.

[14] Khani SC, Karoukis AJ, Young JE, Ambasudhan R, Burch T, Stockton R (2003). Late-onset autosomal dominant macular dystrophy with choroidal neovascularization and nonexudative maculopathy associated with mutation in the RDS gene. Invest Ophthalmol Vis Sci.

[15] Casillo L, Tricarico S, Contento L, Vingolo EM (2021). Clinical Features, Prognosis, and Long-Term Response to Ranibizumab of Macular CNVs in Pattern Dystrophies Spectrum: A Pilot Study. J Ophthalmol.

[16] Anastasakis A, Goleni F, Livir-Rallatos G, Livir-Rallatos C, Zafirakis P, Allen Fishman G (2016). Spontaneous Regression of Choroidal Neovascularization in a Patient with Pattern Dystrophy. Case Rep Ophthalmol Med.

[17] Parodi MB (2002). Choroidal neovascularization in fundus pulverulentus. Acta Ophthalmol Scand.

[18] Parodi MB, Liberali T, Pedio M, Francis PJ, Piccolino FC, Fiotti N (2006). Photodynamic therapy of subfoveal choroidal neovascularization secondary to reticular pattern dystrophy: three-year results of an uncontrolled, prospective case series. Am J Ophthalmol.

[19] Chan WM, Lai TY, Liu DT, Lam DS (2007). Intravitreal bevacizumab (Avastin) for myopic choroidal neovascularization: six-month results of a prospective pilot study. Ophthalmology.

[20] Wiegand TW, Rogers AH, McCabe F, Reichel E, Duker JS (2009). Intravitreal bevacizumab (Avastin) treatment of choroidal neovascularisation in patients with angioid streaks. Br J Ophthalmol.

[21] Parodi MB, Iacono P, Cascavilla M, Zucchiatti I, Kontadakis DS, Bandello F (2010). Intravitreal bevacizumab for subfoveal choroidal neovascularization associated with pattern dystrophy. Invest Ophthalmol Vis Sci.

[22] Nangia P, Shah D, Saurabh K, Roy R (2019). Efficacy of anti-VEGF in the treatment of choroidal neovascular membrane secondary to pattern dystrophy simulating fundus flavimaculatus. GMS Ophthalmol Cases.

[23] Gallego-Pinazo R, Dolz-Marco R, Pardo-López D, Arevalo JF, Díaz-Llopis M (2011). Primary intravitreal ranibizumab for adult-onset foveomacular vitelliform dystrophy. Graefes Arch Clin Exp Ophthalmol.

[24] Empeslidis T, Vardarinos A, Deane J, Banerjee S (2012). Intravitreal ranibizumab in the treatment of butterfly-shaped pattern dystrophy associated with choroidal neovascularization: a case report. Case Rep Ophthalmol.

